# The Application of Quantitative ^1^H-NMR for the Determination of Orlistat in Tablets

**DOI:** 10.3390/molecules22091517

**Published:** 2017-09-10

**Authors:** Shanshan Sun, Mengxia Jin, Xia Zhou, Jinghua Ni, Xiangju Jin, Hongyue Liu, Yinghong Wang

**Affiliations:** State Key Laboratory for Bioactive Substances and Functions of Natural Medicines and Beijing Key Laboratory of New Drug Mechanisms and Pharmacological Evaluation Study, Institute of Materia Medica, Chinese Academy of Medical Sciences and Peking Union Medical College, No.1 Xiannongtan Street, Beijing 100050, China; sunshanshan@imm.ac.cn (S.S.); jinmengxia91@163.com (M.J.); xiazhou@imm.ac.cn (X.Z.); nijh@imm.ac.cn (J.N.); jxj@imm.ac.cn (X.J.); hyl@imm.ac.cn (H.L.)

**Keywords:** qNMR, Orlistat, internal standard method, methodology validation

## Abstract

A quantitative nuclear magnetic resonance (qNMR) method to measure the content of Orlistat in tablets was studied and found to be efficient, accurate, reliable, and simple. In this paper, phloroglucinolanhydrous and dimethylsulfoxide-*d*_6_ (DMSO-*d*_6_) served as the internal standard and solvent, respectively. The qNMR methodology, including the linearity, range, the limit of detection (LOD) and quantification (LOQ), stability, precision, and accuracy, was validated seriatim, and the results were very favorable. The content determination results of three batches of Orlistat in tablets were almost identical upon comparing the qNMR method and the high-performance liquid chromatography (HPLC) method. The recommended method authentically compensated the deficiencies of the current HPLC method for determining Orlistat content, and proved to be a method complementary to traditional analysis for the purity measurement of Orlistat in some pharmaceutical preparations.

## 1. Introduction

First used to determine compound purity and drug content in 1963 [1,2], NMR has since gradually developed into a precise quantitative analysis method. Though it has a lower sensitivity relative to HPLC and mass spectrometry (MS) [3], the quantitative NMR (qNMR) method possesses distinctive advantages: (1) it can be conducted without analyte reference materials; (2) it can provide structural information and does not destroy the samples; (3) it can undertake multicomponent analysis in a mixture without pre-isolation; and (4) it requires a comparatively short time [4,5,6,7,8,9]. Nowadays, qNMR is mainly applied to identify and quantify drugs, biological metabolites, and natural products [10,11,12,13,14,15,16]. The basis of qNMR is the proportional relationship between the given integral resonance and the number of protons. Therefore, the drug content can be calculated with the integral values of the analyte and the internal standard in the same solution. To obtain accurate analysis results, the quantitative peaks and internal standard peaks should not exhibit interference with any other signals. There should also be better optimized spectral acquisition parameters.

**Figure 1:**
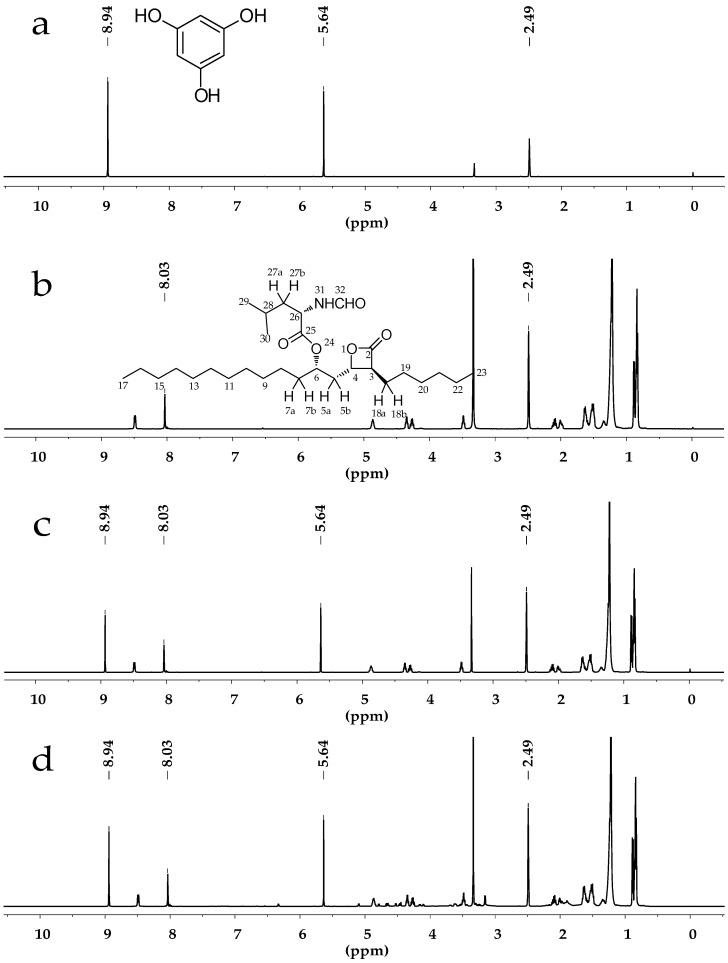
(**a**) ^1^H-NMR spectrum of phloroglucinolanhydrous in dimethylsulfoxide-*d*_6_ (DMSO-*d*_6_); (**b**) ^1^H-NMR spectrum of Orlistat in DMSO-*d*_6_; (**c**) ^1^H-NMR spectrum of Orlistat and phloroglucinolanhydrous in DMSO-*d*_6_; (**d**) ^1^H-NMR spectrum of Orlistat tablet powder extractive and phloroglucinolanhydrous in DMSO-*d*_6_.

Orlistat,(*S*)-((*S*)-1-((2*S*,3*S*)-3-Hexyl-4-oxooxetan-2-yl)tridecan-2-yl)2-formamido-4-methylpentanoate, the structure of which is shown in Figure 1b, is a potent and long-lasting gastrointestinal tract lipase inhibitor that works by directly blocking the fat absorption into bodies. It is currently the sole over-the-counter (OTC) weight-loss drug in the world. The reported methods on Orlistat quantitative analysis in tablets, capsule, and functional foods have mainly been obtained through reversed phase high-performance liquid chromatography (RP-HPLC) [17], HPLC-MS [18], ultra-high-pressure liquid chromatography (UPLC) [19] and microwave-assisted extraction/high-performance liquid chromatography tandem mass spectrometry [20]. These methods not only need more analysis time and consume large quantities of mobile phase, but also require rigorous experimental conditions and imply complicated operational procedures. However, no research on qNMR used to determine Orlistat content has yet been reported.

This study found a specific, accurate, efficient, and feasible qNMR method to determine Orlistat content in tablets. Phloroglucinolanhydrous was selected as the internal standard to mix with Orlistat in dimethylsulfoxide-*d*_6_ (DMSO-*d*_6_). The practicability of the qNMR method was further verified by the consistent determination results of three batches of Orlistat tablets using the qNMR method and the HPLC method.

## 2. Results and Discussion

### 2.1. Selection of Deuterated Solvent

Several deuterated solvents were screened for the experiment. Chloroform-*d*_1_ was abandoned because its strong volatility, which made the solution volume variable, increased the difficulty in quantification. Deuterium oxide was excluded on account of its poor solubility for Orlistat (less than 1 mg/100 mL at 23 °C). Methanol-*d*_4_ was unsuitable because the signals of phenolic hydroxyl protons of phloroglucinolanhydrous did not appear. Acetone-*d*_6_ was eliminated due to the volatility and the close signal interference (shown in Supplementary Materials Figure S5). DMSO-*d*_6_ was an excellent solvent, as it ensured a good solubility of Orlistat in DMSO-*d*_6_ of about 19 mg/mL, and as the quantitative signals of Orlistat and phloroglucinolanhydrous in DMSO-*d*_6_ did not overlap with other signals. Moreover, DMSO-*d*_6_ does not volatilize at room temperature.

All displayed chemical shifts were calibrated relative to the signals of DMSO-*d*_6_ at 2.49 ppm. The quantitative proton of Orlistat was selected at 8.03 ppm (singlet, H-32) while quantitative protons of phloroglucinolanhydrous were selected at 5.64 ppm (singlet, Ph-H) and 8.94 ppm (singlet, OH). The NMR spectra are exhibited in Figure 1a,b. The solvent signals at 2.49 ppm did not interfere with the analyte signal at 8.03 ppm or the internal standard signals at 5.64 and 8.94 ppm.

### 2.2. Determination of Relaxation Time

The main influencing factor in qNMR is the relaxation time, which depends on the longitudinal relaxation time (T1) of all signals during the acquisition. T1 is calculated with the following Equation (1):

(1) $$M_{z}=M_{0}(1-e^{-(\tau /T1)})$$

*M*_z_ and *M*_0_ belong to the magnetizations along the z-axis. Spin-lattice relaxation occurs and reaches a thermal equilibrium after the repetition time (τ) [9]. τ should not be less than five times the T1 to ensure a reliable experimental data [21].

In this work, the inversion recovery pulse sequence experiment was used to determine the T1 of all the protons in the mixture solution of Orlistat (9.028 mg/mL) and phloroglucinolanhydrous (1.019 mg/mL). The relaxation delay used for the inversion recovery experiments was 32 s. The T1 of Orlistat at 8.03 ppm was 1.7 s and the T1 of phloroglucinolanhydrous at 5.64 and 8.94 ppm was 2.2 s and 1.5 s, respectively. According to the preceding standpoint, the relaxation delay of 32s was enough to ensure absolutely T1 relaxation between any two neighbor pulses.

### 2.3. Validation

#### 2.3.1. Specificity and Selectivity

The specificity and selectivity were to estimate the possible interference from sample solutions. According to the solution preparation processes, a specificity study was carried out through analyzing DMSO-*d*_6_, phloroglucinolanhydrous, Orlistat standard solution, and the sample solution individually. ^1^H-NMR spectra are presented in Figure 1. It was clear that the solvent and ingredients did not affect signals at δ 8.03, 5.64 and 8.94 ppm after the extraction. Moreover, the three integrated signals did not overlap each other. The spectra revealed a satisfying specificity and selectivity of the qNMR method for Orlistat determination.

#### 2.3.2. Linearity and Range

Linearity was assessed by measuring six different concentration solutions of Orlistat in the range (*w*/*w*) of 1.075, 3.076, 6.039, 9.079, 11.765, 16.971. The calibration curves are presented below with the ratio of the mass as the x-axis and integral values as the y-axis. The correlation coefficients of quantitative protons at 5.64 ppm in Linear Equation (2) and 8.94 ppm in Linear Equation (3) were 0.99996 and 0.99997, respectively. The regression results showed a perfect linearity of the qNMR method.

(2) y=0.08400x+0.00238

(3) y=0.08343x+0.00300

#### 2.3.3. Accuracy

The accuracy of qNMR was evaluated by the recovery test, in which a known quantity of Orlistat (at 80%, 100%, and 120%, respectively) was added into the tablet powder after extraction. The accuracy was calculated with Equation (4), shown below. The average recoveries were 99.83% and 99.45% with the relative standard deviations (RSDs) of 1.46% and 1.50%, respectively. Table 1 suggests that the qNMR method could provide an ideal accuracy for Orlistat determination.

(4) $$Recovery(\%)=\frac{m_{x}-m_{0}}{m_{s}}\times 100\%$$

*m_x_* is the obtained weight of Orlistat, *m*_0_ is the Orlistat weight in tablets, and *m_s_* is the weight of the added standard Orlistat.

#### 2.3.4. Precision

Good or poor precision was expressed by the RSD of repeatability and intra-day precision. The repeatability was tested using three different concentration solutions in triplicate, and the intra-day precision was tested with the Orlistat concentration at 8.246 mg/mL on different days. The RSDs for both repeatability and intra-day precision, shown in Table 2, were less than 1%, which satisfied the requirement of the Chinese Pharmacopoeia.

#### 2.3.5. Stability

The stability further showed whether there was significant variation after the initial solution was stored for a period of time. The stability of Orlistat solution at a concentration of 8.316 mg/mLwas assessed by comparing the amount of Orlistat present in the initial sample versus the sample stored for 0, 6, 12, 24, 48, and 72 h at room temperature, as shown in Table 3. The RSDs were 0.19% and 0.14% for δ 5.64 and 8.94 ppm, respectively, which indicated that the Orlistat solution was adequately stable during the testing period.

#### 2.3.6. Limit of Detection (LOD) and Limit of Quantification (LOQ)

The concentration was defined as LOD when the signal-to-noise ratio (S/N) of quantitive signals reached 3:1 or 2:1. Similarly, it was defined as LOQ when the S/N was 10:1. This study found that the LOD for Orlistat in DMSO-*d*_6_ was about 0.004 mg/mL (S/N was 2.98:1) and the LOQ was 0.014 mg/mL (S/N was 9.71:1).

#### 2.3.7. Robustness

Robustness refers to the effect of experimental results caused by the variations of acquisition parameters. The robustness assessment of this study was based on the difference analysis from the single variable test of the following acquisition parameters: the number of scans, the relaxation delay, the pulse width, the data points, spectral width, the acquisition time, and selected different integrated protons. The Orlistat content (about 8.246 mg/mL) measured with optimal parameters was 100.46% and 99.67% at δ 5.64 and 8.94 ppm, respectively. Robustness results are exhibited in Table 4. The maximum difference of 0.57% illustrated that these parameters did not significantly alter the results in comparison to the optimized state.

### 2.4. Assay of Orlistat in Tablets

The proposed method was exploited to determine Orlistat content in three batches of tablets, and the qNMR results were compared with the HPLC results. The average values of %label claim (*n* = 3) and RSD% are listed in Table 5. The determination results at 5.64 ppm were higher than those at 8.94 ppm, perhaps due to the difference between the T1 of the two protons. The determined contents using the qNMR and HPLC methods were almost identical, which indicated that qNMR could develop into a method for purity determination parallel to the conventional methods.

## 3. Materials and Methods

### 3.1. Materials

The Orlistat reference material 99.3% (standard for HPLC) was bought from National Institutes for Food and Drug Control (Batch No.: 520027-201401). The commercial Orlistat tablets were bought from ZHE JIANG HISUN PHARMACEUTICAL CO., LTD. (Zhejiang, China; Batch No. 15082101, 0.12 g; Batch No. 21606151, 0.12 g; and Batch No. 21605111, 0.12 g). The 99.0% phloroglucinolanhydrous was obtained from TCI (Shanghai, China) Development Co., Ltd. (Shanghai, China; CN NO.: 61727; Lot. JAM8I-EN, 25 g). Deuterated solvent (DMSO-*d*_6_, 99.9%) was bought from Sigma-Aldrich Co., (Louis, MO, USA). Methanol (HPLC Grade) was bought from Fisher Scientific (Fair Lawn, NJ, USA). The Wahaha water (596 mL, Batch No.: 52157W) was bought from the Wahaha Group Co., Ltd. (Tianjin, China).

### 3.2. Instruments

All NMR data were obtained with a Bruker Spectrometer (AV-III-500, Burlingame, CA, USA) equipped with Cryoprobes (5 mm CPPTCI 1H/19F-13C/15N/D z-GRD z135420/0004) at 500.06 MHz proton frequency. All the solid substances were weighed with METTLER TOLEDO XP2U (0.0001 mg, Greifensee, Switzerland).

HPLC was performed on a Shimadzu Prominence LC-20A liquid chromatography system with an SPD-M20A prominence diode array detector and Inertsil ODS-3 (5 μm, 4.6 mm × 250 mm, Shimadzu Co., Kyoto, Japan).

### 3.3. Test Solutions Preparation

#### 3.3.1. Standard Solution Preparation for qNMR

A moderate amount of Orlistat reference material (about 1, 3, 6, 9, 12, and 18 mg) and phloroglucinolanhydrous (about 1 mg) were accurately weighed and transferred into a stoppered tube, and then 1 mL of DMSO-*d*_6_ was added to fully dissolve the two materials with the ultrasound. The transparent liquid was transferred into a 5-mm NMR tube, and then the data acquisition was carried out. Each concentration solution was prepared in triplicate.

#### 3.3.2. Tablet Powder Solution Preparation for qNMR

Ten commercial Orlistat tablets were weighed and powdered in a mortar. Then, proper Orlistat tablet powder (equivalent to Orlistat 10 mg) was weighed and extracted three times by chloroform. When the chloroform fully volatilized at room temperature, the next step was performed similarly to that described in Section 3.3.1.

#### 3.3.3. Sample Solution Preparation for HPLC

The Orlistat reference material (about 1 mg) was weighed accurately and fully dissolved in 2 mL of methanol. Then, it was filtered into a vial for HPLC analysis with 0.45 μm Nylon membrane. This solution was used as the standard solution. The Orlistat tablet powder (equivalent to Orlistat 1 mg) was prepared using the same method. 

### 3.4. Data Acquisition and Processing

The optimal experiment parameters for ^1^H-NMR spectra at 298.0 K are listed below: pulse angle 90°, pulse width 8.00 μs, data points 64 K, number of scans 32, relaxation delay 32 s (five times as much as T1 to ensure full relaxation), acquisition time (AQ) 3.277 s, and spectral width (SW) 20.00 ppm.

For all ^1^H-NMR obtained spectra, the manual corrections of the phase and baseline were essential to ensure correct, accurate, and repeatable integrations of the typical quantitative peaks and internal standard peaks. To satisfy the statistic, each spectrum was manually integrated five times and the average value was used for calculation.

For HPLC, the mobile phase consisted of methanol and 1‰ formic acid aqueous solution with a constant-gradient ratio of 92:8. The chromatographic condition included a temperature of 30 °C, flow rate of 1.5 mL/min, injection volume of 20 μL, and ultraviolet (UV) detection at 210 nm.

### 3.5. Content Calculation

As has been reported in the literature, the fundamental principle of qNMR is that the NMR signals intensity (*I*) has a direct ratio relation with the number of nuclei (*n*) [8,9], as the following Equation (5) describes:

(5) I=Ks×n

*Ks* is an unknown constant to all proton signals in the same ^1^H-NMR spectrum because they are running the same single-pulse. Although different signals contain different numbers of nuclei, their relationship can be described by Equation (6):

(6) $$\frac{I_{1}}{I_{2}}=\frac{n_{1}}{n_{2}}$$

The the percentage of Orlistat accounting for tablets label claim can be calculated by Equation (7):

(7) $$\%LC=\frac{Ix}{Istd}\frac{Nstd}{Nx}\frac{Mx}{Mstd}\frac{mstd}{mpowder}\times \frac{T}{L}\times Pstd\times 100\%$$

*%LC* is the percentage of Orlistat accounting for tablets label claim. *Ix* and *Istd* indicate the integrated value of Orlistat and phloroglucinolanhydrous. *Nx* and *Nstd* define the number of protons contained in the quantitative signals of Orlistat and phloroglucinolanhydrous. *Mx* and *Mstd* correspond to the molecular weight of Orlistat and phloroglucinolanhydrous. *mpowder* and *mstd* denote the mass of Orlistat tablet powder and phloroglucinolanhydrous. *T* is the average tablet weight; and *L* is the labeled amount of Orlistat in the tablets. *Pstd* represents the purity of phloroglucinolanhydrous.

## 4. Conclusions

A practicable and reliable qNMR method was developed and evaluated to measure the content of Orlistat in tablets. The content determination results of qNMR were consistent with HPLC, and proved that qNMR was a specific, accurate, precise, simple, and repeatable method for Orlistat content determination. The proposed qNMR method authentically perfected the determination analysis method of Orlistat, which is currently mainly measured by HPLC. The qNMR method is simpler in multicomponent sample preparation, faster in sample analysis, less rigorous in experimental conditions, and better in repeatability compared with HPLC. The most vital advantage of the qNMR method over traditional analytical methods is that it can achieve precise quantification without analyte reference materials; thus, the qNMR method makes it possible for medicine and compounds that lack reference materials to be absolutely quantified. The content determination results of Orlistat tablets was almost identical when comparing the qNMR method and HPLC method, which indicates that the qNMR method could be used in a complementary manner to the traditional analysis method for the purity measurement of Orlistat in some pharmaceutical preparations.

## Figures and Tables

**Table 1:** Recovery test results of Orlistat purity determination.

No.	Orlistat				Recovery (%)	
	*m*_0_ (mg)	*m_s_* (mg)	*m_x_* (mg)			
			δ*5.64	δ*8.94	δ*5.64	δ*8.94
1	2.302	1.795	4.096	4.091	99.94	99.68
2	2.238	1.943	4.136	4.136	97.71	97.71
3	2.295	1.875	4.170	4.154	100.03	99.19
4	2.259	2.197	4.442	4.442	99.35	99.39
5	2.311	2.271	4.556	4.527	98.85	97.57
6	2.248	2.295	4.507	4.497	98.43	97.97
7	2.099	2.768	4.873	4.896	100.20	101.02
8	2.229	2.853	5.136	5.114	101.89	101.13
9	2.213	2.821	5.093	5.073	102.07	101.36
Average value	/	/	/	/	99.83	99.45
RSD%	/	/	/	/	1.46	1.50

δ* in ppm; /: blank cell; RSD: relative standard deviations; *m_x_*: obtained weight of Orlistat; *m*_0_: weight of Orlistat in tablets; *m_s_*: weight of added standard Orlistat.

**Table 2:** Precision and repeatability results of Orlistat purity determination.

	No.	*mstd* (mg)	*m_x_* (mg)	*Px* (%)	
				δ*5.64	δ*8.94
Repeatability	1	1.119	6.042	98.91	99.16
	2	1.068	6.024	99.09	98.14
	3	1.072	6.050	98.83	97.87
	4	1.122	9.343	98.64	99.25
	5	1.092	9.060	98.96	98.53
	6	1.067	8.833	98.11	97.10
	7	1.078	11.164	98.49	97.83
	8	1.055	12.930	99.08	98.16
	9	1.118	11.200	98.65	97.82
	Average value	/	/	98.75	98.21
	RSD %	/	/	0.32	0.70
Inter-day precision	1	/	/	100.40	99.65
	2			100.46	99.68
	3			100.43	99.62
	4			100.35	99.61
	5			100.20	99.32
	6			100.10	99.49
	Average value	/	/	100.32	99.56
	RSD %	/	/	0.14	0.14

δ* in ppm; /: blank cell; *mstd*: weight of phloroglucinolanhydrous; *m_x_*: weight of Orlistat; *Px*: purity of Orlistat.

**Table 3:** Stability results of Orlistat purity determination.

Time (h)	δ*5.64		δ*8.94	
	Assay (%)	Diff (%)	Assay (%)	Diff (%)
0	100.30	/	99.53	/
6	100.14	0.16	99.37	0.16
12	100.07	0.23	99.26	0.28
24	100.01	0.29	99.23	0.31
48	99.90	0.40	99.24	0.30
72	99.74	0.60	99.12	0.41
Average value	100.03	/	99.30	/
RSD %	0.19	/	0.14	/

δ* in ppm; /: blank cell.

**Table 4:** Robustness results of Orlistat purity determination.

Parameters (Target Value)	Change	δ*5.64		δ*8.94	
		Assay (%)	Diff (%)	Assay (%)	Diff (%)
Number of scans (32)	16	100.11	0.35	99.39	0.28
	48	100.05	0.41	99.33	0.34
Relaxation delay (32 s)	24	100.08	0.38	99.36	0.31
	40	99.98	0.47	99.37	0.30
Acquisition time (3.277 s)	2.277	100.13	0.33	99.55	0.12
	4.277	100.21	0.25	99.51	0.16
Data points (64 K)	32	100.03	0.43	99.43	0.25
	128	99.93	0.53	99.44	0.23
Spectral width (20 ppm)	15	99.89	0.57	99.35	0.32
	25	99.91	0.55	99.36	0.31
P1 (8.00 μsec)	7.00	99.95	0.51	99.30	0.35
	9.00	99.96	0.50	99.40	0.27

δ* in ppm.

**Table 5:** The determination results of Orlistat by qNMR and HPLC.

Batch No.	qNMR (*n* = 3)			HPLC	
	δ	% Label Claim	RSD%	% Label Claim	RSD%
15082101	5.64	100.65	0.38	99.71	0.61
	8.94	99.99	0.34		
21606151	5.64	98.58	1.11	96.75	0.85
	8.94	99.03	0.86		
21605111	5.64	98.75	0.33	97.87	1.20
	8.94	97.42	0.51		

*δ in ppm.

## Supplementary Materials

The following are available online, Figure S1: ^1^H-NMR spectrum of Orlistat in DMSO-*d*_6_ (500 MHz), Figure S2: COSY-NMR spectrum of Orlistat in DMSO-*d*_6_ (500 MHz), Figure S3: TOCSY-NMR spectrum of Orlistat in DMSO-*d*_6_ (500 MHz), Figure S4: ROESY-NMR spectrum of Orlistat in DMSO-*d*_6_ (500 MHz), Figure S5: ^1^H-NMR spectrum of Orlistat and Phloroglucinol Anhydrous in different deuterated solvents, Table S1:The assignment of ^1^H-NMR data of the Orlistat in DMSO-*d*_6_, Table S2: The integral values of different experiments.
