# Supplementary material for: The Application of Quantitative 1H-NMR for the Determination of Orlistat in Tablets
Source: Molecules. 2017 Sep 10;22(9):1517. doi: 10.3390/molecules22091517 (PMC6151513; doi:10.3390/molecules22091517)
Supplement: Supplementary file 1 [file molecules-22-01517-s001.zip › molecules-220456-supplementary/Table S1. The assignment of 1H-NMR data of the Orlistat in DMSO-d6.docx]

**Table S1.** The assignment of ^1^H-NMR data of the Orlistat in DMSO-d_6_

| Position | δH (J／Hz) | ^１^H-^１^H COSY |
| --- | --- | --- |
| 31 | 8.483-8.498 (7.64, 1H, d) | H-32, H-26 |
| 32 | 8.034 (1H, s) | H-31 |
| 6 | 4.860-4.866 (1H, m) | H-5a, H-5b, H-7a, H-7b |
| 4 | 4.346-4.354 (1H, m) | H-3, H-5a, H-5b |
| 26 | 4.261-4.274 (1H,m) | H-31, H-27a, H-2b |
| 3 | 3.333-3.496 (1H, m) | H-4, H-18a, H-18b |
| 5a | 2.084-2.098 (1H, m) | H-6, H-4, H-5b |
| 5b | 1.999-2.012 (1H, m) | H-6, H-4, H-5a |
| 28, 18a,18b | 1.593-1.656 (3H, m) | H-3, H-27a, H-27b |
| 7a, 7b, 27a, 27b | 1.485-1.547 (4H, m) | H-6, H-26 |
| 8, 9, 10, 11, 12, 13, 14, 15, 16, 19, 20, 21, 22, | 1.219-1.348 (26H, m) | * |
| 17, 23, 29, 30 | 0.828-0.891 (12H, m) | * |
|  |  |  |
|  |  |  |
|  |  |  |

* means undefined correlations of protons

**References**

1. Chen, Z.X.; Yu, S.T.; Xia, Z.J. Orlistat organic spectral analysis. *Chinese　Journal　of　Magnetic　Resonance.* **2013**, 30, 585-593.
