# Supplementary material for: The Application of Quantitative 1H-NMR for the Determination of Orlistat in Tablets
Source: Molecules. 2017 Sep 10;22(9):1517. doi: 10.3390/molecules22091517 (PMC6151513; doi:10.3390/molecules22091517)
Supplement: Supplementary file 1 [file molecules-22-01517-s001.zip › molecules-220456-supplementary/Table S2. The integral values of different enperiments.docx]

**Table S2.** The integral values of different enperiments

| **Linearity and Range at 5.64 ppm** |
| --- |

| *NO.* | *Ix1* | *Ix2* | *Ix3* | *Ix4* | *Ix5* | *Istd* |
| --- | --- | --- | --- | --- | --- | --- |
| 1 | 0.0900 | 0.0901 | 0.0901 | 0.0900 | 0.0900 | 1 |
| 2 | 0.2550 | 0.2551 | 0.2553 | 0.2552 | 0.2552 | 1 |
| 3 | 0.4713 | 0.4713 | 0.4715 | 0.4714 | 0.4711 | 1 |
| 4 | 0.7002 | 0.7008 | 0.7012 | 0.7011 | 0.7008 | 1 |
| 5 | 0.9198 | 0.9202 | 0.9201 | 0.9200 | 0.9204 | 1 |
| 6 | 1.3736 | 1.3743 | 1.3747 | 1.3749 | 1.3747 | 1 |

| **Recovery test at 5.64 ppm** | |
| --- | --- |
| \| *NO.* \| *Ix1* \| *Ix2* \| *Ix3* \| *Ix4* \| *Ix5* \| *Istd* \| \| --- \| --- \| --- \| --- \| --- \| --- \| --- \| \| *1* \| *0.7005* \| *0.7007* \| *0.7015* \| *0.7014* \| *0.7014* \| *1* \| \| *2* \| *0.7025* \| *0.7012* \| *0.7013* \| *0.7019* \| *0.7019* \| *1* \| \| *3* \| *0.7136* \| *0.7145* \| *0.7146* \| *0.7147* \| *0.7145* \| *1* \| \| *4* \| *0.7608* \| *0.76* \| *0.7601* \| *0.7602* \| *0.7601* \| *1* \| \| *5* \| *0.7729* \| *0.7728* \| *0.7736* \| *0.773* \| *0.7725* \| *1* \| \| *6* \| *0.7651* \| *0.7639* \| *0.7649* \| *0.7654* \| *0.7642* \| *1* \| \| *7* \| *0.8269* \| *0.826* \| *0.827* \| *0.827* \| *0.8269* \| *1* \| \| *8* \| *0.8409* \| *0.8412* \| *0.8439* \| *0.8433* \| *0.8432* \| *1* \| \| *9* \| *0.834* \| *0.8334* \| *0.8364* \| *0.8366* \| *0.8366* \| *1* \| |  |
|  | |

**Repeatability at 5.64 ppm**

| *NO.* | *Ix1* | *Ix2* | *Ix3* | *Ix4* | *Ix5* | *Istd* |
| --- | --- | --- | --- | --- | --- | --- |
| 1 | 0.4579 | 0.4575 | 0.4576 | 0.4576 | 0.4567 | 1 |
| 2 | 0.7031 | 0.7035 | 0.7035 | 0.704 | 0.7038 | 1 |
| 3 | 0.8731 | 0.8739 | 0.8736 | 0.8739 | 0.8739 | 1 |
| 4 | 0.4784 | 0.4786 | 0.4791 | 0.4788 | 0.4788 | 1 |
| 5 | 0.7027 | 0.7028 | 0.7038 | 0.7038 | 0.7032 | 1 |
| 6 | 1.0395 | 1.0408 | 1.0399 | 1.0395 | 1.0408 | 1 |
| 7 | 0.4775 | 0.4779 | 0.4778 | 0.4778 | 0.4778 | 1 |
| 8 | 0.6949 | 0.696 | 0.6963 | 0.6956 | 0.6955 | 1 |
| 9 | 0.8468 | 0.846 | 0.8467 | 0.8466 | 0.8464 | 1 |

| **inter-day precision at 5.64 ppm** |
| --- |
| \| *NO.* \| *Ix1* \| *Ix2* \| *Ix3* \| *Ix4* \| *Ix5* \| *Istd* \| \| --- \| --- \| --- \| --- \| --- \| --- \| --- \| \| 1 \| 0.6825 \| 0.6838 \| 0.6845 \| 0.684 \| 0.684 \| 1 \| \| 2 \| 0.6838 \| 0.6836 \| 0.6844 \| 0.6844 \| 0.6844 \| 1 \| \| 3 \| 0.6831 \| 0.6831 \| 0.6845 \| 0.6845 \| 0.6846 \| 1 \| \| 4 \| 0.6818 \| 0.6818 \| 0.6844 \| 0.6844 \| 0.6845 \| 1 \| \| 5 \| 0.6798 \| 0.6787 \| 0.6844 \| 0.6844 \| 0.6845 \| 1 \| \| 6 \| 0.6803 \| 0.6803 \| 0.6826 \| 0.6826 \| 0.6826 \| 1 \| |
| **Stability at 5.64 ppm** |
| \| *Time (h)* \| *Ix1* \| *Ix2* \| *Ix3* \| *Ix4* \| *Ix5* \| *Istd* \| \| --- \| --- \| --- \| --- \| --- \| --- \| --- \| \| 0 \| 0.6883 \| 0.6882 \| 0.6892 \| 0.6893 \| 0.6893 \| 1 \| \| 6 \| 0.6874 \| 0.6875 \| 0.6881 \| 0.6879 \| 0.688 \| 1 \| \| 12 \| 0.6865 \| 0.6868 \| 0.6878 \| 0.6876 \| 0.6876 \| 1 \| \| 24 \| 0.6861 \| 0.6861 \| 0.6873 \| 0.6873 \| 0.6875 \| 1 \| \| 48 \| 0.6858 \| 0.686 \| 0.6861 \| 0.6861 \| 0.6864 \| 1 \| \| 72 \| 0.6849 \| 0.6849 \| 0.6849 \| 0.6852 \| 0.6852 \| 1 \| |
| **Robustness at 5.64 ppm** |

| *Parameters (target value)* | *Ix1* | *Ix2* | *Ix3* | *Ix4* | *Ix5* | *Istd* |
| --- | --- | --- | --- | --- | --- | --- |
| Acquisition time(2.277s) | 0.6817 | 0.682 | 0.682 | 0.6818 | 0.6819 | 1 |
| Acquisition time(4.277s) | 0.6825 | 0.6822 | 0.6824 | 0.6824 | 0.6826 | 1 |
| Data points (128 K) | 0.6808 | 0.6802 | 0.6803 | 0.6806 | 0.6806 | 1 |
| Data points (32 K) | 0.6812 | 0.6812 | 0.6814 | 0.681 | 0.681 | 1 |
| Number of scans(16) | 0.6817 | 0.6818 | 0.6816 | 0.6817 | 0.6819 | 1 |
| Number of scans(48) | 0.6809 | 0.6815 | 0.6816 | 0.6813 | 0.6814 | 1 |
| P1 (7.00μsec) | 0.6806 | 0.6806 | 0.6806 | 0.6806 | 0.6809 | 1 |
| P1 (9.00μsec) | 0.6807 | 0.6808 | 0.6807 | 0.6807 | 0.6807 | 1 |
| Relaxation delay (24s) | 0.6815 | 0.6815 | 0.6815 | 0.6816 | 0.6816 | 1 |
| Relaxation delay (40s) | 0.6809 | 0.6808 | 0.6809 | 0.6808 | 0.6811 | 1 |
| Spectral width (15 ppm) | 0.6804 | 0.6804 | 0.6803 | 0.6801 | 0.68 | 1 |
| Spectral width (25 ppm) | 0.6801 | 0.6807 | 0.6807 | 0.6804 | 0.6802 | 1 |

**Assay of Orlistat in tablets at 5.64 ppm**

| \| Batch No. \| *Ix1* \| *Ix2* \| *Ix3* \| *Ix4* \| *Ix5* \| *Istd* \| \| --- \| --- \| --- \| --- \| --- \| --- \| --- \| \| 15082101-1 \| 0.8956 \| 0.896 \| 0.8968 \| 0.8968 \| 0.8969 \| 1 \| \| 15082101-2 \| 0.9038 \| 0.9035 \| 0.9042 \| 0.9047 \| 0.9045 \| 1 \| \| 15082101-3 \| 0.9254 \| 0.9252 \| 0.9253 \| 0.9251 \| 0.9251 \| 1 \| \|  \|  \|  \|  \|  \|  \|  \| \| 201605111-1 \| 0.8637 \| 0.8599 \| 0.863 \| 0.8601 \| 0.8739 \| 1 \| \| 201605111-2 \| 0.9466 \| 0.9483 \| 0.9498 \| 0.9469 \| 0.9452 \| 1 \| \| 201605111-3 \| 0.9592 \| 0.9579 \| 0.9579 \| 0.966 \| 0.9611 \| 1 \| \|  \|  \|  \|  \|  \|  \|  \| \| 201606151-1 \| 0.8837 \| 0.8835 \| 0.8835 \| 0.8834 \| 0.8801 \| 1 \| \| 201606151-2 \| 0.8744 \| 0.8742 \| 0.8742 \| 0.8714 \| 0.8743 \| 1 \| \| 201606151-3 \| 0.8941 \| 0.8936 \| 0.8931 \| 0.8935 \| 0.8941 \| 1 \| |
| --- | --- | --- | --- | --- | --- | --- | --- | --- | --- | --- | --- | --- | --- | --- | --- | --- | --- | --- | --- | --- | --- | --- | --- | --- | --- | --- | --- | --- | --- | --- | --- | --- | --- | --- | --- | --- | --- | --- | --- | --- | --- | --- | --- | --- | --- | --- | --- | --- | --- | --- | --- | --- | --- | --- | --- | --- | --- | --- | --- | --- | --- | --- | --- | --- | --- | --- | --- | --- | --- | --- | --- | --- | --- | --- | --- | --- | --- | --- | --- | --- | --- | --- | --- | --- |

| **Linearity and Range at 8.94 ppm** |
| --- |

| *NO.* | *Ix1* | *Ix2* | *Ix3* | *Ix4* | *Ix5* | *Istd* |
| --- | --- | --- | --- | --- | --- | --- |
| 1 | 0.0900 | 0.0900 | 0.0857 | 0.0899 | 0.0898 | 1 |
| 2 | 0.2537 | 0.2538 | 0.2539 | 0.2538 | 0.2541 | 1 |
| 3 | 0.4681 | 0.4688 | 0.4686 | 0.4689 | 0.4688 | 1 |
| 4 | 0.6982 | 0.6996 | 0.6992 | 0.6986 | 0.6988 | 1 |
| 5 | 0.9119 | 0.9129 | 0.9126 | 0.9126 | 0.9126 | 1 |
| 6 | 1.3653 | 1.3653 | 1.3668 | 1.3668 | 1.3663 | 1 |

| **Recovery test at 8.94 ppm** |
| --- |
| \| *NO.* \| *Ix1* \| *Ix2* \| *Ix3* \| *Ix4* \| *Ix5* \| *Istd* \| \| --- \| --- \| --- \| --- \| --- \| --- \| --- \| \| 1 \| 0.7 \| 0.7002 \| 0.7005 \| 0.7004 \| 0.7004 \| 1 \| \| 2 \| 0.7015 \| 0.7002 \| 0.702 \| 0.7025 \| 0.7025 \| 1 \| \| 3 \| 0.7106 \| 0.7106 \| 0.7124 \| 0.7124 \| 0.7124 \| 1 \| \| 4 \| 0.7618 \| 0.7602 \| 0.7599 \| 0.76 \| 0.76 \| 1 \| \| 5 \| 0.7683 \| 0.7682 \| 0.7686 \| 0.768 \| 0.7671 \| 1 \| \| 6 \| 0.7626 \| 0.7614 \| 0.7627 \| 0.7633 \| 0.7645 \| 1 \| \| 7 \| 0.8295 \| 0.8303 \| 0.831 \| 0.831 \| 0.8314 \| 1 \| \| 8 \| 0.8362 \| 0.8364 \| 0.8411 \| 0.8405 \| 0.8406 \| 1 \| \| 9 \| 0.8291 \| 0.8329 \| 0.8328 \| 0.8329 \| 0.833 \| 1 \|   **Repeatability at 8.94 ppm** |
| \| *NO.* \| *Ix1* \| *Ix2* \| *Ix3* \| *Ix4* \| *Ix5* \| *Istd* \| \| --- \| --- \| --- \| --- \| --- \| --- \| --- \| \| 1 \| 0.4578 \| 0.459 \| 0.4581 \| 0.4592 \| 0.459 \| 1 \| \| 2 \| 0.7075 \| 0.7095 \| 0.7076 \| 0.7074 \| 0.7075 \| 1 \| \| 3 \| 0.8663 \| 0.8682 \| 0.868 \| 0.8686 \| 0.8678 \| 1 \| \| 4 \| 0.4739 \| 0.4741 \| 0.4743 \| 0.4742 \| 0.4742 \| 1 \| \| 5 \| 0.7002 \| 0.7004 \| 0.7009 \| 0.6996 \| 0.6999 \| 1 \| \| 6 \| 1.0309 \| 1.0306 \| 1.0303 \| 1.0299 \| 1.0305 \| 1 \| \| 7 \| 0.4726 \| 0.4732 \| 0.4733 \| 0.4732 \| 0.4732 \| 1 \| \| 8 \| 0.687 \| 0.6888 \| 0.6891 \| 0.6888 \| 0.689 \| 1 \| \| 9 \| 0.8386 \| 0.8399 \| 0.8395 \| 0.8393 \| 0.8394 \| 1 \| |
| **inter-day precision at 8.94 ppm**   \| *NO.* \| *Ix1* \| *Ix2* \| *Ix3* \| *Ix4* \| *Ix5* \| *Istd* \| \| --- \| --- \| --- \| --- \| --- \| --- \| --- \| \| 1 \| 0.6768 \| 0.6781 \| 0.6798 \| 0.6793 \| 0.6792 \| 1 \| \| 2 \| 0.678 \| 0.6779 \| 0.6794 \| 0.6794 \| 0.6794 \| 1 \| \| 3 \| 0.6774 \| 0.6773 \| 0.6791 \| 0.6792 \| 0.6792 \| 1 \| \| 4 \| 0.6764 \| 0.6764 \| 0.6798 \| 0.6795 \| 0.6795 \| 1 \| \| 5 \| 0.6753 \| 0.6753 \| 0.6772 \| 0.677 \| 0.677 \| 1 \| \| 6 \| 0.675 \| 0.675 \| 0.6793 \| 0.6793 \| 0.6792 \| 1 \| |
| **Stability at 8.94 ppm**   \| *Time (h)* \| *Ix1* \| *Ix2* \| *Ix3* \| *Ix4* \| *Ix5* \| *Istd* \| \| --- \| --- \| --- \| --- \| --- \| --- \| --- \| \| 0 \| 0.6829 \| 0.6829 \| 0.6839 \| 0.6841 \| 0.6841 \| 1 \| \| 6 \| 0.6817 \| 0.6818 \| 0.683 \| 0.6829 \| 0.6829 \| 1 \| \| 12 \| 0.6802 \| 0.6806 \| 0.6826 \| 0.6825 \| 0.6825 \| 1 \| \| 24 \| 0.6804 \| 0.6804 \| 0.6822 \| 0.6822 \| 0.6822 \| 1 \| \| 48 \| 0.6814 \| 0.6816 \| 0.6815 \| 0.6816 \| 0.6816 \| 1 \| \| 72 \| 0.6806 \| 0.6806 \| 0.6807 \| 0.681 \| 0.681 \| 1 \| |

**Robustness at 8.94 ppm**

| \| *Parameters (target value)* \| *Ix1* \| *Ix2* \| *Ix3* \| *Ix4* \| *Ix5* \| *Istd* \| \| --- \| --- \| --- \| --- \| --- \| --- \| --- \| \| Acquisition time(2.277s) \| 0.6778 \| 0.678 \| 0.6781 \| 0.6779 \| 0.6779 \| 1 \| \| Acquisition time(4.277s) \| 0.6779 \| 0.6777 \| 0.6776 \| 0.6776 \| 0.6776 \| 1 \| \| Data points (128 K) \| 0.6776 \| 0.6769 \| 0.6769 \| 0.6773 \| 0.6773 \| 1 \| \| Data points (32 K) \| 0.6773 \| 0.6773 \| 0.6772 \| 0.6768 \| 0.6769 \| 1 \| \| Number of scans(16) \| 0.6767 \| 0.6768 \| 0.6768 \| 0.6769 \| 0.677 \| 1 \| \| Number of scans(48) \| 0.6761 \| 0.6766 \| 0.6767 \| 0.6764 \| 0.6764 \| 1 \| \| P1 (7.00μsec) \| 0.6763 \| 0.6763 \| 0.6764 \| 0.6764 \| 0.6764 \| 1 \| \| P1 (9.00μsec) \| 0.6768 \| 0.677 \| 0.677 \| 0.6769 \| 0.6769 \| 1 \| \| Relaxation delay (24s) \| 0.6765 \| 0.6765 \| 0.6766 \| 0.6767 \| 0.6769 \| 1 \| \| Relaxation delay (40s) \| 0.677 \| 0.6768 \| 0.6767 \| 0.6766 \| 0.6765 \| 1 \| \| Spectral width (15 ppm) \| 0.6766 \| 0.6766 \| 0.6766 \| 0.6765 \| 0.6766 \| 1 \| \| Spectral width (25 ppm) \| 0.6762 \| 0.6768 \| 0.677 \| 0.6767 \| 0.6767 \| 1 \| |
| --- | --- | --- | --- | --- | --- | --- | --- | --- | --- | --- | --- | --- | --- | --- | --- | --- | --- | --- | --- | --- | --- | --- | --- | --- | --- | --- | --- | --- | --- | --- | --- | --- | --- | --- | --- | --- | --- | --- | --- | --- | --- | --- | --- | --- | --- | --- | --- | --- | --- | --- | --- | --- | --- | --- | --- | --- | --- | --- | --- | --- | --- | --- | --- | --- | --- | --- | --- | --- | --- | --- | --- | --- | --- | --- | --- | --- | --- | --- | --- | --- | --- | --- | --- | --- | --- | --- | --- | --- | --- | --- | --- |

**Assay of Orlistat in tablets at 8.94 ppm**

| Batch No. | *Ix1* | *Ix2* | *Ix3* | *Ix4* | *Ix5* | *Istd* |
| --- | --- | --- | --- | --- | --- | --- |
| 15082101-1 | 0.8888 | 0.8921 | 0.8884 | 0.8922 | 0.8916 | 1 |
| 15082101-2 | 0.8964 | 0.898 | 0.8984 | 0.8982 | 0.8967 | 1 |
| 15082101-3 | 0.9187 | 0.9185 | 0.9208 | 0.9206 | 0.9205 | 1 |
|  |  |  |  |  |  |  |
| 201605111-1 | 0.8554 | 0.8547 | 0.8547 | 0.8549 | 0.8549 | 1 |
| 201605111-2 | 0.9371 | 0.9388 | 0.9388 | 0.9359 | 0.9359 | 1 |
| 201605111-3 | 0.9399 | 0.9387 | 0.9447 | 0.9467 | 0.9398 | 1 |
|  |  |  |  |  |  |  |
| 201606151-1 | 0.8893 | 0.889 | 0.889 | 0.8889 | 0.8889 | 1 |
| 201606151-2 | 0.8783 | 0.8781 | 0.8781 | 0.8784 | 0.8784 | 1 |
| 201606151-3 | 0.895 | 0.8945 | 0.8945 | 0.895 | 0.895 | 1 |

|  |
| --- |
